# The impact of an acute high polyphenol, high fiber meal with and without aerobic exercise on metabolism in middle‐aged and older adults: A pilot study

**DOI:** 10.14814/phy2.70312

**Published:** 2025-05-29

**Authors:** L. J. Ater, E. K. Plantz, T. D. Manning, J. D. Akers, E. S. Edwards, S. P. Kurti

**Affiliations:** ^1^ Department of Kinesiology, Human Performance Laboratory James Madison University Harrisonburg Virginia USA; ^2^ Integrated Nutrition and Physiology Laboratory, Department of Health Professions James Madison University Harrisonburg Virginia USA

**Keywords:** acute exercise, fiber, glycemia, lipemia, polyphenols, postprandial

## Abstract

A high fat, high carbohydrate (HFHC) meal can induce adverse triglyceride (TRG), glucose, and metabolic load index (MLI; TRG + glucose) in middle‐aged and older adults. A bout of exercise (EX) or an acute meal may attenuate these postprandial responses. This study aimed to determine whether a high polyphenol, high fiber meal with and without EX could reduce postprandial TRG, glucose, and MLI in this population. In a randomized crossover design, 10 healthy adults (56.9 ± 6.9 years, 6F, 4M) completed four conditions: (1) traditional HFHC, (2) T‐HFHC + EX, (3) HFHC meal with polyphenols and fiber (P‐HFHC), (4) a P‐HFHC + EX. Each participant consumed 12 kcals/kg body mass. The P‐HFHC was made with plant‐based ingredients to match the macronutrient composition of the T‐HFHC. EX, performed 30 min post‐meal, expended 25% of kcals consumed. Blood TRG and glucose were measured for 6 h post‐meal, and MLI was calculated. There was a significant time*condition interaction for TRG (*p* = 0.038), glucose (*p* = 0.001), and MLI (*p* = 0.026). The P‐HFHC condition had lower TRGs at 4 and 5 h (*p* = 0.031, *p* = 0.050). These findings suggest that a minimally processed meal or EX may reduce CVD risk in middle‐aged and older adults.

## 1 INTRODUCTION

Cardiovascular disease and diabetes are leading causes of mortality and morbidity globally (World Health Statistics, 2023). The prevalence of chronic disease can be attributed largely to contemporary dietary patterns and physical inactivity. The frequent, excessive consumption of food, particularly ultra‐processed foods high in saturated fat and added sugar, has detrimental effects on metabolic health. Even a single high‐fat and high‐carbohydrate meal substitute (HFHC) can raise triglyceride (TRG) and glucose levels to a magnitude that impairs endothelial function and contributes to the development of insulin resistance (Vogel et al., 1997). HFHCs amplify oxidative stress/inflammatory‐mediated mechanisms through increases in both TRG and glucose; therefore, it is important to consider their combined increase through the metabolic load index (MLI; TRG + glucose) (Emerson et al., 2016). The frequent consumption of HFHCs results in many individuals spending much of their day in a postprandial state (Keirns et al., 2021). Consequently, postprandial TRG and glucose levels may be a better predictor of risk compared to fasting levels alone (Bansal et al., 2007). Middle‐aged and/or older adults tend to experience higher, more prolonged TRG and glucose responses following a HFHC due to increased fat mass, decreased lean body mass, increased insulin resistance, and decreased lipoprotein lipase (LPL) activity when compared to younger counterparts (Fink et al., 1983; Katsanos, 2014; Vinagre et al., 2018).

Acute aerobic exercise (Gillen et al., 2021; Hardman & Aldred, 1995; Katsanos & Moffatt, 2004; Kurti, Frick, et al., 2022) and nutritional challenges (Feldman et al., 2021; Gruendel et al., 2006; Khossousi et al., 2008; Lee et al., 2020) have individually been shown to attenuate post‐prandial lipemia (PPL) and post‐prandial glycemia (PPG) following a HFHC meal. Polyphenols, which are naturally occurring bioactive compounds found in plant‐based foods (i.e., berries, dark chocolate, flax seeds, coffee), have been investigated to lower acute post‐prandial responses because of their antioxidant properties. Supplementation with polyphenols may lower post‐prandial TRG and glucose through increasing lipid clearance by inhibiting digestive enzymes such as α‐amylase and α‐glucosidase, thereby improving insulin sensitivity (Nyambe‐Silavwe & Williamson, 2016), and also reducing oxidative stress and inflammation (Edirisinghe et al., 2011). Consuming fiber has also been shown to have beneficial effects on post‐prandial glucose, lipids, and inflammation (O'Keefe et al., 2008). Fiber can reduce carbohydrate and fat absorption by creating viscous gels in the intestine, which alter gut motility and microbiota and ultimately delay fat absorption (Bozzetto et al., 2018). Fiber also decreases gastric emptying (Lee et al., 2020) and produces short‐chain fatty acids (Makki et al., 2018), which ultimately improve glucose homeostasis and reduce TRG synthesis (Dahl & Stewart, 2015).

In addition, it is possible the fiber and polyphenols will have combined effects on TRG and glucose by amplifying insulin sensitivity due to the combination of short‐chain fatty acid production by fiber and polyphenol‐mediated insulin signaling described above. Also, the dual inhibition of pancreatic lipase by polyphenols and fat absorption by fiber may lead to combined benefits on postprandial TRG. Finally, both fiber and polyphenols have reduced oxidative stress and inflammation, further mitigating metabolic dysfunction after a HFHC meal (Nyambe‐Silavwe & Williamson, 2016). However, few studies have investigated the combined effects of polyphenols, fiber plus EX in mitigating the metabolic response to a HFHC meal.

Although pre‐prandial exercise (EX) has been shown to have the most substantial effect on reducing post‐meal metabolic outcomes (O'Keefe et al., 2008), this research aimed to investigate whether a low energy expenditure single bout of acute aerobic EX, suitable for incorporation after a single meal and into an adults' daily routine, could offer similar benefits. Numerous studies have investigated the effects of acute post‐prandial EX on TRG and/or glucose, including accumulated EX near meals (Zhang et al., 2022), high‐intensity interval activity (Gay et al., 2018), or moderate‐intensity continuous EX (De Nardi et al., 2018). However, results have been inconsistent, likely due to differences in EX intensity, duration, participant demographics, and EX timing (Katsanos & Moffatt, 2004). While the literature remains conflicting, moderate‐intensity EX for 20–30 min, starting at 30 min after a meal, appears effective in blunting glucose surges (Chacko, 2016) and is a practical dose for daily life. To our knowledge, no study has investigated the combined effects of multiple HFHC meals with and without EX in a controlled trial; specifically in healthy middle‐aged and older adults. The present study evaluated whether the combination of acute EX and a high polyphenol, high fiber, minimally processed = (NOVA scale ≤3 and no food additives) meal (P‐HFHC) attenuates PPL, PPG, and MLI in middle‐aged and older adults to a greater extent than either acute intervention alone. We hypothesized that both the acute nutritional challenge and acute EX would lower PPL, PPG, and MLI; however, the largest attenuation would be in the P‐HFHC + EX condition.

## 2 METHODS

### 2.1 Participants

Ten healthy middle‐aged and older adults (56.9 ± 6.9 years, 4M, 6F) were recruited from James Madison University and Harrisonburg, VA, through bulk email and word of mouth. Participants were given a Physical Activity Questionnaire (PAR‐Q+) to assess whether they were free from CVD, metabolic and renal disease, and could participate in the EX‐trial without physician approval. Participants were excluded if they were not cleared for exercise from the PAR‐Q+. Participants were included if they were not consuming antioxidant supplements, anti‐inflammatory agents, or medications that could potentially interfere with the outcomes (e.g., statins, anti‐hypertensives, anti‐diabetic agents, etc.). Additionally, all participants provided verbal and written consent. The descriptive characteristics of the participants are shown in Table 2. The research was approved by the James Madison University Institutional Review Board #22–3437.

### 2.2 Anthropometry and blood pressure

During the initial visit, each subject's height was measured with a portable stadiometer (Charder Model HM 200P, Charder Electronic Co Ltd., Taichung, Taiwan) with shoes removed. Body mass was determined using a standard physician's scale (Dymo Pelouze model 4040, Newell Brands, Hoboken, NJ). Body mass index was calculated from that data. Total body composition, lean body mass, and body fat were measured with a dual‐energy x‐ray absorptiometry (DEXA) scan (GE Lunar iDXA, Fairfield, CT). Following these measurements, participants sat upright for 5 min before their brachial artery blood pressure (BP) was taken with an automatic sphygmomanometer (ProBP 3400 Welch Allyn, Skaneateles Falls, NY). The procedure followed the American College of Sports Medicine (ACSM) guidelines, in which two blood pressure measurements were taken and averaged (Liguori et al., 2021).

### 2.3 Experimental design

Participants visited the Human Performance Lab at James Madison University to complete four different conditions. The order of these conditions was randomized using a random number generator. The conditions were assigned numbers 1 through 4 as follows: 1) a traditional HFHC challenge alone (T‐HFHC) previously shown to increase inflammation and metabolic outcomes (Emerson et al., 2018, 2019; Kurti, Frick, et al., 2022; Kurti, Wisseman, et al., 2022) (Vogel et al., 1997); 2) HFHC challenge followed by a bout of exercise (T‐HFHC + EX) (Emerson et al., 2016); 3) a redesigned HFHC meal substitute composed of high polyphenol, high fiber, and minimally processed ingredients (P‐HFHC) (Keirns et al., 2021); 4) P‐HFHC followed by a bout of exercise (P‐HFHC + EX). Each condition was separated by at least 72 h with no longer than 3 weeks between trials. Participants refrained from any EX for 48 h prior to all conditions. All HFHC challenges were consumed in the same time frame for each subject, and each was completed between the hours of 6 am and 10 am. The participants were unaware of which HFHC meal condition they received. Body mass was assessed during each visit to the lab to ensure that the subject's weight remained stable. Participants were instructed to record their food intake for 24 h before the first HFHC meal challenge and replicate the same diet prior to each subsequent HFHC challenge. Upon arrival at the laboratory, investigators confirmed adherence to the dietary requirements by reviewing the participants' 24‐h recall logs.

### 2.4 Postprandial exercise bout

In the EX‐trials, an acute bout of EX was performed 30 min after the ingestion of the T‐HFHC or the P‐HFHC. The participants exercised at a self‐selected speed and grade on a treadmill to expend 25% of the kilocalories (kcal) consumed from the HFHC (Equation 1). The ACSM walking equation was used to calculate VO_2_ and estimate kilocalorie expenditure (Liguori et al., 2021).

(1) $${\mathrm{VO}}_{2}=\left(0.1\times \text{Speed}\right)+\left(1.8\times \text{Speed}\;x\;\text{Grade}\right)+3.5$$

### 2.5 HFHC challenges

Participants came to the laboratory after an overnight fast. Investigators ensured participants adhered to the prior meal instructions when they arrived at the laboratory. Fingerstick blood samples were taken upon arrival to obtain baseline TRG, glucose, total cholesterol (TC), and HDL‐C measurements. LDL‐C was calculated using the Friedewald equation. Participants had 20 min to consume the meal challenge during each visit. The 6‐h session began at time ‘0’, which was marked by the last bite of the meal. Each meal was standardized to 12 kcal per kg of body mass. Subsequent fingerstick blood samples were taken every 30 min postprandially for 2 h, after which they were taken hourly for up to 6 h. TRG, total cholesterol (TC), LDL‐C, and HDL‐C were assessed at baseline and every hour postprandial. Blood glucose was assessed at baseline and every 30 min for 2 h, then hourly. The blood was analyzed with a Cholestech LDX analyzer (Alere Inc., Waltham, MA). The Cholestech LDX analyzer has high reproducibility when compared to the laboratory gold standard analysis, with intra‐class correlation coefficients exceeding 0.75 for all lipid categories (Dale et al., 2008).

### 2.6 Nutritional conditions

The T‐HFHC and P‐HFHC meal substitutes can be found in Table 3. The T‐HFHC meal substitute was Marie Callender's Chocolate Satin Pie (58% saturated fat, 38% carbohydrate [Conagra Brands, Chicago, IL]). The P‐HFHC was a minimally processed pie made with plant‐based ingredient; designed by a dietitian to not be different in macronutrients when compared to the Marie Callender's Chocolate Satin Pie (fat, CHO, and protein). To blind the participants to which condition they were consuming, the P‐HFHC was matched to the T‐HFHC in visual aesthetics, texture, taste profile, and kcals. Total fat, carbohydrates, and protein were not meant to be different between conditions. The minimally processed ingredients were high in polyphenols (total pie >5000 mg) and dietary fiber (total pie = 45 g). Rather than using animal sources of saturated fat, coconut‐derived plant‐based sources were used. The polyphenols were sourced primarily from cacao powder, flax meal, coconut, and chia seeds.

### 2.7 Statistical analysis

The number of participants necessary was determined by a priori sample size calculation using previous research with the difference in postprandial TRG between the T‐HFHC alone and T‐HFHC + EX condition as the main outcome (Kurti, Frick, et al., 2022). With a power level of 0.80 and a two‐sided alpha of 0.05, eight participants were required per group to observe differences in TRG between the T‐HFHC alone and the T‐HFHC + EX condition. Data were analyzed by IBM SPSS Statistics v29.0 (IBM Corporation, Armonk, NY). All the data collected was analyzed for skewness, kurtosis, and normality using the Shapiro–Wilk test. Data were normally distributed, and repeated‐measures ANOVAs were run to determine whether there were any changes in main outcome measures (TRG, glucose, MLI) and exploratory outcomes (TC, HDL‐C, and LDL‐C) as a main effect of time (baseline to 6 h postprandially) or across the exercise (EX) and diet conditions (T‐HFHC alone or P‐HFHC alone). Dunnett's multiple comparisons test were performed for significant interaction effects to assess pairwise comparisons between the T‐HFHC and P‐HFHC conditions. Secondary analyses for total and incremental area under the curve (t‐AUC, i‐AUC), peak, and time to peak (TTP) were calculated for TRG, Glucose, and MLI using GraphPad Prism (GraphPad Software, Inc) and a one‐way RM‐ANOVA was run to determine whether there were differences between conditions across 6 h. Effect sizes (Cohen's *d*) were calculated for t‐AUC and peak TRG, as well as t‐AUC glucose and MLI, using the means and pooled standard deviations of each condition. Significance was set a priori at *p* < 0.05.

## 3 RESULTS

### 3.1 Participant, EX, and meal characteristics

Participant characteristics can be seen in Table 1 and EX and meal characteristics are found in Table 2. EX energy expenditure ranged from 199.9 to 308.4 kcals, % grade ranged from 1 to 5, and speed ranged from 3.0 to 3.6 mph.

**TABLE 1:** Subject Characteristics.

	Mean ± SD
Age (yrs)	56.9 ± 6.9
Height (cm)	170.4 ± 5.7
Weight (kg)	80.1 ± 13.9
BMI (kg/m^2^)	27.4 ± 3.5
Total Body Fat (%)	36.4 ± 5.7
Android Body Fat (%)	46.8 ± 6.8
Systolic blood pressure (mmHg)	118.6 ± 11.7
Diastolic blood pressure (mmHg)	76.7 ± 8.5

Abbreviation: BMI, body mass index.

**TABLE 2:** Exercise and meal characteristics (mean values and standard deviation).

	Mean ± SD
Exercise duration (mins)	36.8 ± 5.9
Grade (%)	3.5 ± 1.1
Speed (mph)	3.1 ± 0.4
Exercise expenditure (kcals)	240.4 ± 41.7
Meal size (kcals)	961.5 ± 166.8

Meal characteristics of the T‐HFHC and the P‐HFHC are shown in Table 3. The meals were designed to be similar in calories, fat, carbohydrate, and protein content. Due to the use of plant‐based whole food ingredients to match the total fat content between the P‐HFHC and T‐HFHC, the P‐HFHC had significantly higher saturated fat content (*p* < 0.05). The amount consumed by participants was relative to their BW (12 kcal/kg BW).

**TABLE 3:** Meal Characteristics.

	T‐HFHC	P‐HFHC	*p*‐Value
	Mean ± SD	Mean ± SD	
Total kcal	961.5 ± 166.8	961.5 ± 166.8	1.00
Total fat (g)	66.3 ± 11.5	74.8 ± 13.0	0.17
Sat. fat (g)	38.7 ± 6.7	54.0 ± 9.4^a^	<0.01
CHO (g)	84.8 ± 14.7	76.5 ± 13.3	0.23
Sugar (g)	60.8 ± 10.6	51.8 ± 9.0	0.07
Fiber (g)	5.5 ± 1.0	13.2 ± 2.3^a^	<0.01
Protein (g)	9.2 ± 1.6	10.6 ± 1.8	0.11

Abbreviations: CHO, carbohydrate; kcal, kilocalories; sat., saturated.

^a^ Significantly different from the T‐HFHC.

### 3.2 Fasting levels

Fasting levels are shown in Table 4. There was no condition effect on day‐to‐day fasting TRG, glucose, TC, HDL‐C, LDL‐C levels (all *p*'s >0.05).

**TABLE 4:** Fasting levels (mg/dL) prior to each condition (mean values and standard deviation).

	T‐ HFHC	T‐HFHC+EX	P‐HFHC	P‐HFHC+EX	*p*‐Value
	Mean ± SD	Mean ± SD	Mean ± SD	Mean ± SD	
TRG	119.9 ± 15.1	102 ± 9.2	112.8 ± 17.5	103.3 ± 12.4	0.396
GLUCOSE	97.5 ± 9.6	97.5 ± 10.8	100.1 ± 7.2	97.6 ± 11	0.551
LDL‐C	116.6 ± 11.4	116.1 ± 9.7	114.4 ± 10.6	116.8 ± 12.5	0.365
HDL‐C	50.8 ± 3.9	51.8 ± 4.5	52.5 ± 3.9	55.9 ± 3.9	0.752
TC	191.4 ± 14.5	188.3 ± 11.5	189.5 ± 14.2	193.3 ± 13.3	0.105

### 3.3 Time‐course of postprandial metabolic response across conditions

**FIGURE 1:**
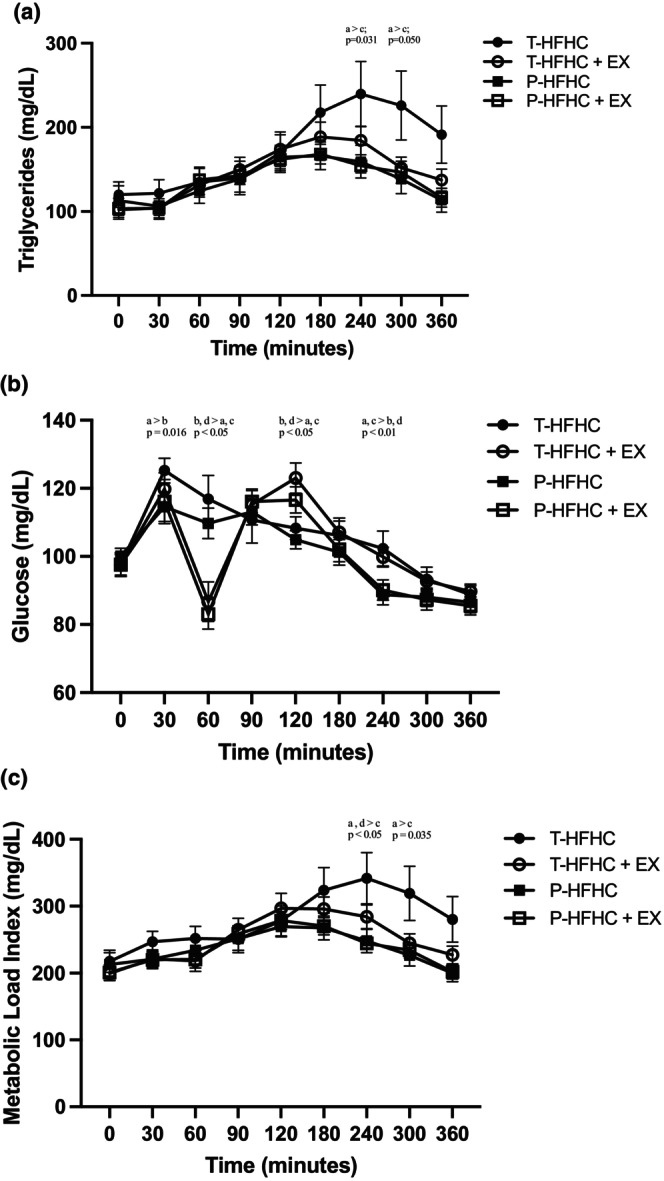
(a) TRG; (b) Glucose; and (c) MLI in each acute HFHC meal condition with and without EX. Data are presented as mean ± SE. ^a,b,c,d^Denotes differences across the following four conditions, respectively, for a given time point. Traditional high‐fat high‐carbohydrate meal with and without EX; T‐HFHC, TFHC + EX, respectively; and high‐polyphenol and fiber HFHC with and without exercise; P – HFHC, P – HFHC + EX, respectively.

Postprandial metabolic responses for TRG, glucose, and MLI can be seen in Figure 1a–c. There was a significant increase in TRG from baseline to 6 h postprandially across all conditions (*p* < 0.001). There was a significant interaction between TRG and condition (*p* = 0.038). Specifically, the TRG response was significantly greater during the T‐HFHC condition compared to the P‐HFHC at 4 h (*p* = 0.031) and 5 h (*p* = 0.050). There was a trend towards a lower TRG response in the P – HFHC + EX at 4 h compared to the T‐HFHC (*p* = 0.09).

There was a significant change in glucose across time (*p* < 0.01), between conditions (*p* = 0.007), and as an interaction between time and condition (*p* = 0.001). The P‐HFHC had a lower glucose response at 30 min postprandially compared to T‐HFHC (*p* = 0.016). At an hour postprandially, the T‐HFHC and P‐HFHC were higher than the T‐HFHC + EX and the P‐HFHC + EX (all *p*'s <0.050). At 2 h post‐prandially, the T‐HFHC and P‐HFHC were significantly lower than the EX‐conditions (all *p*'s <0.010). At 4 h postprandially, the T‐HFHC alone and T‐HFHC + EX were higher than the P‐HFHC alone and the P‐HFHC + EX (*p* < 0.050), but T‐HFHC was not different than the T‐HFHC + EX (*p* = 0.828).

MLI was significant across time (*p* < 0.001) and as an interaction between time and condition (*p* = 0.026). Specifically, the P‐HFHC alone was significantly lower than the T‐HFHC alone at 4 h (*p* = 0.020) and 5 h (*p* = 0.035) post‐prandially. The P‐HFHC + EX was also lower than the T‐HFHC at 4 h post‐prandially (*p* = 0.049).

**FIGURE 2:**
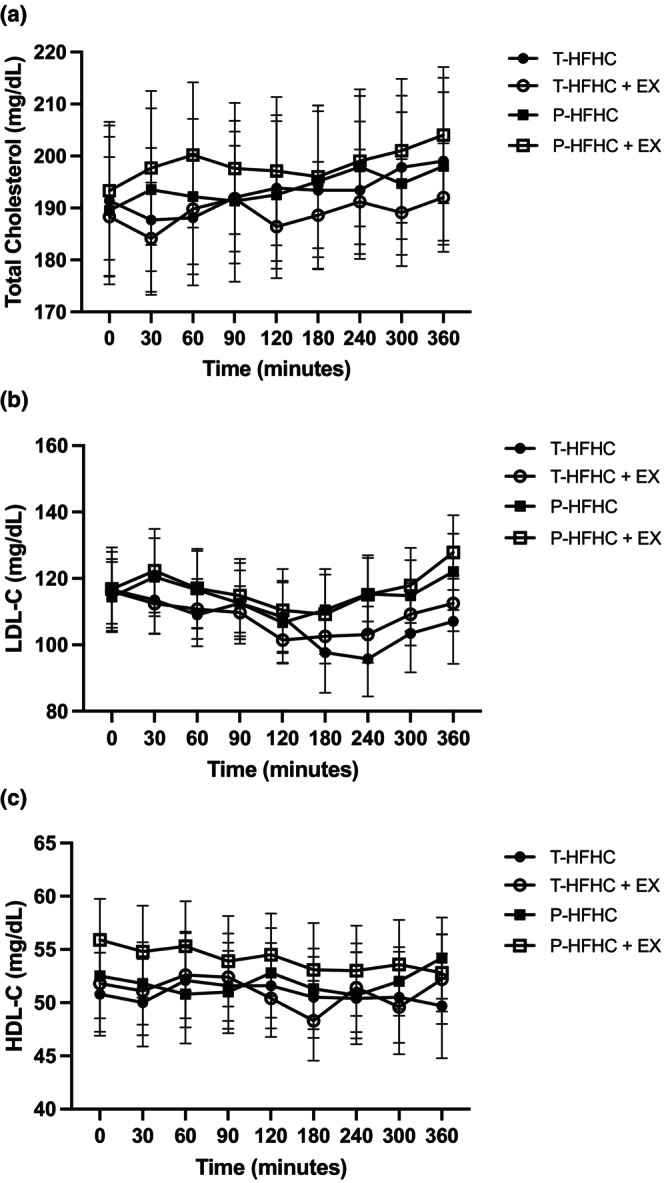
Data are presented as mean and standard error. (a) TC; (b) LDL‐C, and (c) HDL‐C in each cute HFHC meal condition with and without EX. There were no significant interaction effects across time between the four conditions (Traditional high‐fat high‐carbohydrate meal with and without EX; T‐HFHC, TFHC + EX, respectively; and high‐polyphenol and fiber HFHC with and without exercise; P – HFHC, P – HFHC + EX, respectively).

Postprandial metabolic responses for TC, HDL‐C, and LDL‐C can be seen in Figure 2a–c. There was no significant difference in TC, HDL‐C, and LDL‐C by condition (*p* = 0.919, *p* = 0.934, *p* = 0.853, respectively), or as an interaction between time and condition for TC, HDL‐C, or LDL‐C (all *p*'s >0.05).

### 3.4 Postprandial lipid, glucose, and MLI responses across conditions

**FIGURE 3:**
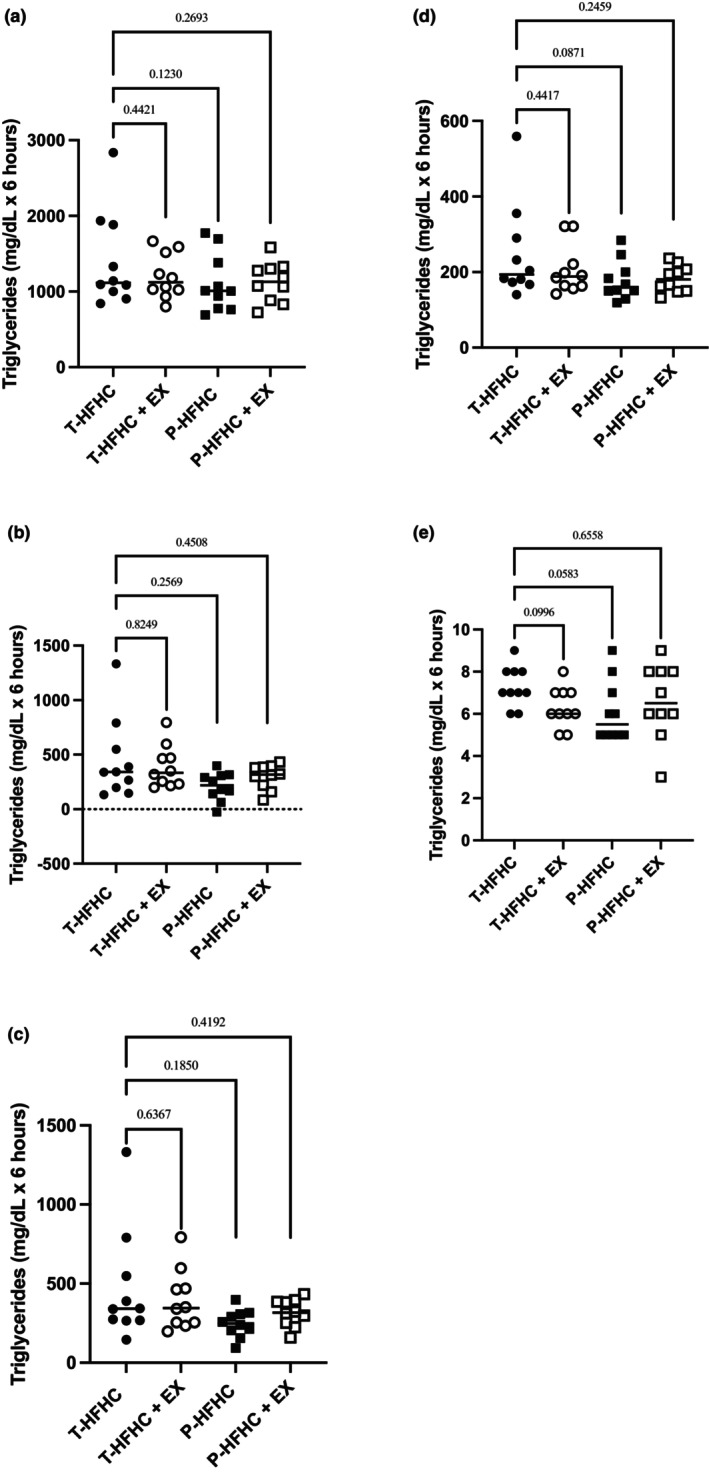
Individual TRG data is shown with mean values displayed across the entire post‐prandial period. (a) TRG total AUC; (b) iAUCnet; (c) iAUCtotal; (d) Peak; (e) time to peak (TTP). *p*‐values are displayed compared to the T‐HFHC.

**FIGURE 4:**
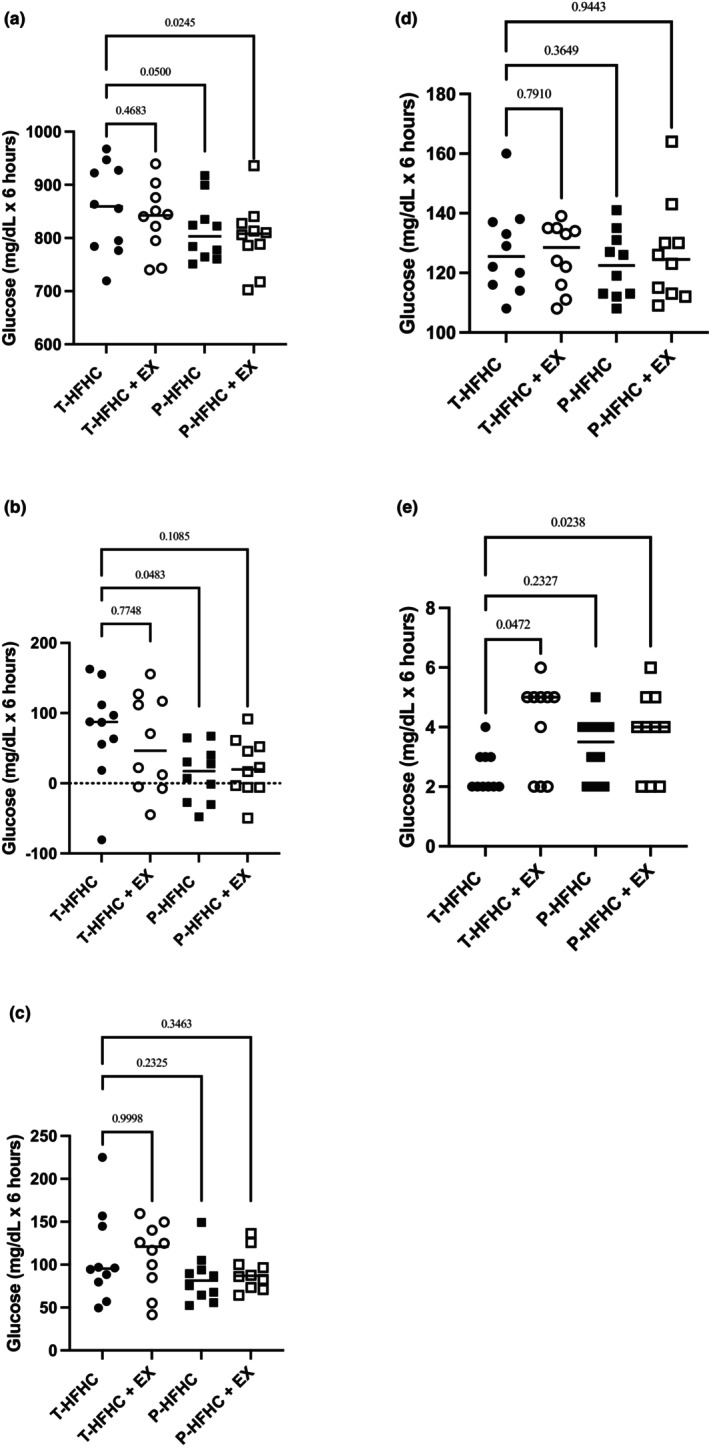
Individual GLU data is shown with mean values displayed across the entire post‐prandial period. (a) TRG total AUC; (b) iAUCnet; (c) iAUCtotal; (d) Peak; (e) time to peak (TTP). *p*‐values are displayed compared to the T‐HFHC.

**FIGURE 5:**
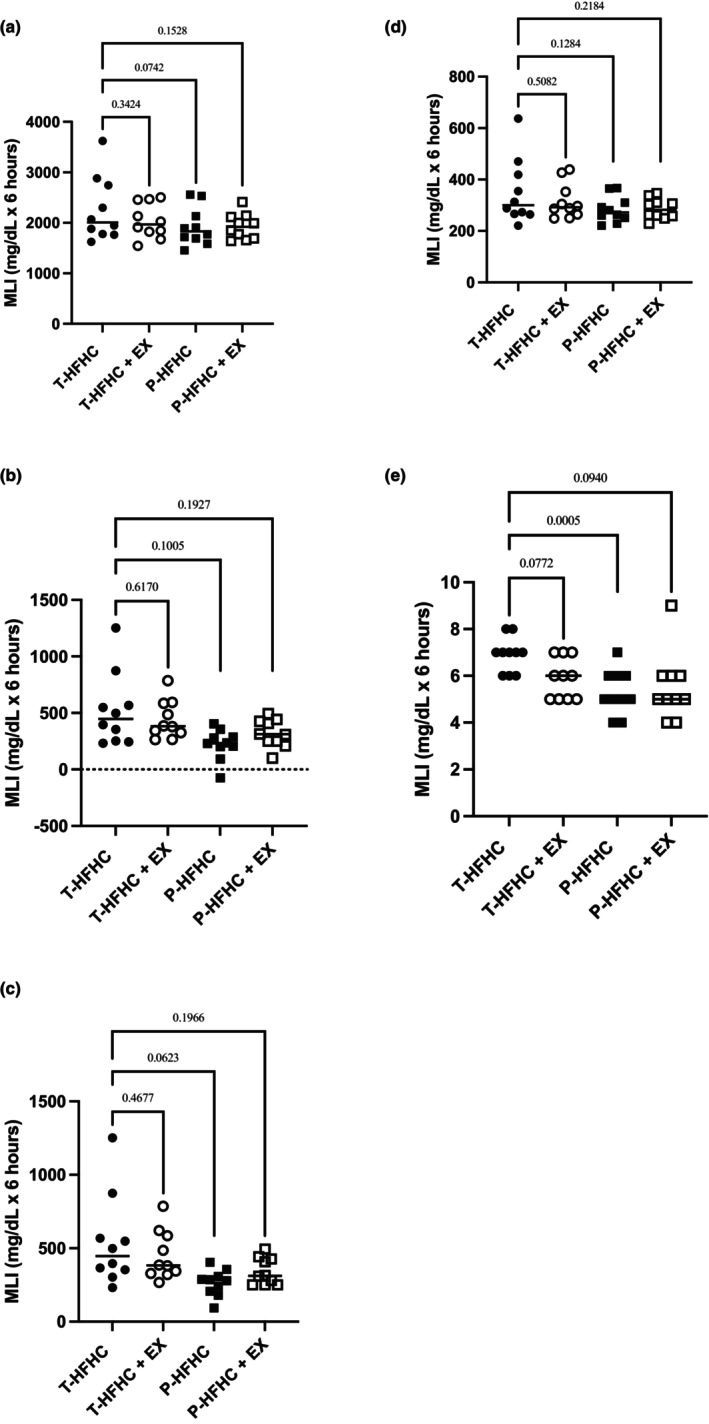
Individual MLI data is shown with mean values displayed across the entire post‐prandial period. (a) TRG total AUC; (b) iAUCnet; (c) iAUCtotal; (d) Peak; (e) time to peak (TTP). *p*‐values are displayed compared to the T‐HFHC.

Postprandial t‐AUC, iAUCnet, and iAUCtotal, peak responses, and TTP are displayed with p‐values in Figure 3a–e for TRG, Figure 4a–e for glucose, and Figure 5a–e for MLI. Although no statistically significant differences were observed for TRG t‐AUC, several pairwise comparisons yielded small or moderate effect sizes. The T‐HFHC elicited a greater response than the T‐HFHC + EX (Cohen's *d* = 0.41), P‐HFHC (Cohen's *d* = 0.56) and P – HFHC + EX (Cohen's *d* = 0.58). For peak TRG, The T‐HFHC elicited a greater response than the T‐HFHC + EX (Cohen's *d* = 0.42), P‐HFHC (Cohen's *d* = 0.72) and P – HFHC + EX (Cohen's *d* = 0.71).

Although there were significant differences in glucose t‐AUC, several pairwise comparisons demonstrated small and moderate effect sizes. The T‐HFHC elicited a greater glucose response compared to T‐HFHC + EX (Cohen's *d* = 0.27), P‐HFHC (Cohen's *d* = 0.58) and P – HFHC + EX (Cohen's *d* = 0.71). A similar trend was observed for MLI, where T‐HFHC + EX showed a small to moderate effect (Cohen's *d* = 0.43), and P‐HFHC and P‐HFHC + EX produced moderate effects compared to T‐HFHC (Cohen's *d* = 0.65 and 0.69, respectively).

## 4 DISCUSSION

In the present study, consuming a P‐HFHC meal as well as the combination of T‐HFHC + EX and a P‐HFHC + EX attenuated postprandial glucose responses compared to a T‐HFHC meal. Additionally, there was a moderate effect size for the reduction in TRGs with P‐HFHC and P‐HFHC + EX, indicating a substantial impact on lipid metabolism. This finding confirms previous literature that the combination of diet and EX may be effective in reducing postprandial TRG and glucose (Davis et al., 2020; Polley et al., 2019). However, contrary to our hypothesis, P‐HFHC + EX did not attenuate TRG and glucose to a greater extent than either acute intervention alone. Interestingly, P‐HFHC alone had the greatest attenuation at specific time points for TRG, whereas P‐HFHC and P‐HFHC had a greater effect on glucose and MLI. These findings support the use of dietary strategies (minimally processed foods with polyphenol and fiber) and acute EX to minimize the adverse effects of a HFHC meal.

### 4.1 Postprandial lipid responses and the impact of exercise

Our study does not support previous findings that EX after the consumption of a T‐HFHC attenuates the postprandial TRG response (Hardman & Aldred, 1995; Katsanos & Moffatt, 2004). In the present study, EX beginning 30 min after a HFHC meal, expending 25% of the kcals consumed, did not significantly reduce the TRG response in healthy adults. The EX conditions demonstrated a small to moderate effect in reducing TRG levels compared to the T‐HFHC meal. Interestingly, the energy expenditure used in this study elicited similar reductions in TRGs compared to recent work from our laboratory where participants expended 75% of the kcals consumed from a HFHC (Kurti, Frick, et al., 2022). The TRG reduction reported in the present study compared to previous work from our laboratory does contrast with existing literature on energy expenditure and the postprandial TRG response, which suggests that the magnitude of energy expended directly determines the magnitude of attenuation (Gill et al., 2002; Katsanos, 2006; Zhang et al., 2007). However, differences in participant descriptives may explain TRG reductions. In the current study, the participants had a higher peak TRG response by ~40 to 50 mg/dL and greater visceral adiposity (~6%) compared to those in the previous study. This difference is significant because individuals with higher visceral adiposity tend to have larger postprandial TRG responses and thus may experience a greater attenuation from acute EX (Plaisance & Fisher, 2014). While most sources suggest that energy expenditures greater than 500 kcals are required to see the TRG‐lowering effects of acute EX (Katsanos, 2006; Plaisance & Fisher, 2014), the participants in the current study expended an average of 240 ± 41.7 kcals. Despite this lower energy expenditure, most participants had TRG levels reduced below the 220 mg/dL threshold in the T‐HFHC + EX condition, a level considered clinically significant when evaluating postprandial TRG responses (Kolovou et al., 2011). The participants with the largest TRG responses saw the greatest blunting effect from EX that brought them from high‐risk levels to normal or lower‐risk values. Specifically, four participants had TRG responses greater than 220 mg/dL in the T – HFHC condition. These same participants all saw reductions in TRG below this threshold with 25% energy expenditure in the P – HFHC + EX. Three of the four participants had reductions below this threshold in the T – HFHC + EX condition. This shows that even though the P‐HFHC and P‐HFHC + EX seemed to have a greater effect on postprandial TRG, EX had an effect in individuals with adverse postprandial responses and it is likely increasing the intensity or duration would elicit larger TRG reductions.

There are multiple proposed mechanisms in which aerobic EX would attenuate the postprandial TRG response. The most well‐known mechanism is through an increase in LPL activity, although the literature is conflicting about whether that is the primary mechanism acting in post‐meal exercise conditions (Katsanos, 2006; Petridou & Mougios, 2022). The increase in LPL activity has been shown to take effect 4–18 h after exercise (Plaisance & Fisher, 2014; Seip et al., 1997). This aligns with the timing seen in the current study, in which there was a trend towards a reduction at 4 h after EX (*p* = 0.08). When exercise is performed after the ingestion of a HFHC, the increased blood flow may allow for increased contact between TRG and LPL, providing the opportunity for TRG to be hydrolyzed (Klein et al., 1992; Teeman et al., 2016a). The research has also shown that moderate‐intensity exercise close to meal ingestion results in greater intramuscular TRG utilization (Romijn et al., 1993). The depletion of intramuscular TRG increases the clearance of circulating TRG by skeletal muscle LPL (Katsanos, 2006). Finally, there may have been enhanced post‐exercise insulin sensitivity (Petitt & Cureton, 2003). After an acute bout of EX, insulin sensitivity increases and remains elevated for at least 16 h (Borghouts & Keizer, 2000). Increased insulin sensitivity has been linked with promoting the clearance of TRG from circulation by increasing TRG storage in adipose tissue (Burton‐Freeman et al., 2010).

### 4.2 The impact of exercise on postprandial glucose responses

Our findings suggest that exercise performed 30 min after a HFHC meal, expending as low as 25% of the kcals consumed, is sufficient in attenuating the postprandial glucose response. The results confirm previous literature stating that exercise 30 min post‐meal is effective in blunting peak glucose values (Chacko, 2016). The processes by which exercise lowers postprandial glucose are beyond the scope of this study; however, possible mechanisms at play include an increase in blood flow to the skeletal muscle and GLUT‐4 translocation via activation of the AMPK pathway (Musi et al., 2003; Richter et al., 2017; Webster et al., 2010).

Interestingly, in both EX conditions, glucose increased at 120 min postprandially. While it is not uncommon for a post‐exercise, post‐meal increases in glucose, the magnitude seen in this study is larger than in our previous work in young adults expending 50% of the calories consumed in a high‐fat meal (Teeman et al., 2016b). However, four participants in the current study had impaired fasting glucose, which research has shown can lead to altered glucose responses (Melton et al., 2009). Furthermore, the increase in glucose levels at 120 min in the exercise conditions may be a result of delayed glucose absorption (Wang et al., 2018). Further research is needed to understand this occurrence mechanistically.

### 4.3 The use of a high fiber, high polyphenol, minimally processed meal substitute to lower post‐prandial TRG, glucose, and MLI

Both P‐HFHC and P‐HFHC + EX elicited a reduction in metabolic outcomes following a HFHC. A finding consistent with previous research shows that acute polyphenol and fiber nutritional strategies can reduce the postprandial TRG (Feldman et al., 2021; Khossousi et al., 2008; Lee et al., 2020) and glucose (Boers et al., 2017; Coe & Ryan, 2016; Kawakami et al., 2021) responses to a HFHC. A meta‐analysis by Feldman et al. reported that acute polyphenol intake positively impacts postprandial outcomes, most notably the TRG response (Feldman et al., 2021). Across 14 studies, a 5%–39% decrease in TRG was observed. Comparably, in the current study, a 31% decrease in peak TRG was observed with the P‐HFHC meal. The effects of fiber and polyphenols on the magnitude of reduction in TRG have been shown to act in a dose‐dependent manner, with higher amounts of fiber exhibiting greater lipid reductions compared to lower amounts of fiber (Gruendel et al., 2006).

The FDA recommends an intake of 1000 mg of polyphenols a day for general health benefits and a fiber intake of 25–38 g per day (Institute of Medicine, 2005). However, an acute dose of polyphenols effective in reducing postprandial TRG has not been established due to the complexity of considering quality, composition, bioavailability, and method of delivery (Feldman et al., 2021). The dosage of supplemented polyphenols used in previous studies ranges from 150 to 3000 mg (Feldman et al., 2021). In studies using healthy participants, the polyphenol doses, 196, 600, and 948 mg respectively, did not produce significant effects (Mathew et al., 2012; Ochiai et al., 2015; Richter et al., 2017) whereas, a study looking at participants with dyslipidemia saw reductions in TRG with doses as low as 338 mg (Burton‐Freeman et al., 2010). The average intake of food‐derived polyphenols by the participants in the current study was around 1544 mg. Our results indicate that polyphenols can effectively reduce the TRG response in healthy participants when consumed in higher doses, though our meal also was minimally processed and contained a higher fiber content.

The meal challenges were designed to not be statistically different from the T‐HFHC in calories, fat, sugar, and total carbohydrates. However, because of the use of plant‐based products to increase the fat content rather than animal products, saturated fat ended up being significantly higher in the P‐HFHC. Using a plant‐based source rather than an animal‐based source of saturated fat may have blunted the TRG response (Panth et al., 2020). While this was not what we originally intended in the design of the pies, typically an increase in saturated fat would be thought to increase postprandial TRG (Folwaczny et al., 2021) and MLI (Kurti et al., 2023) compared to a meal substitute with lower saturated fat or other types of fatty acids (Masson & Mensink, 2011; Rivellese et al., 2003), though this is not always the case (Monfort‐Pires et al., 2016; Sciarrillo et al., 2019). However, simple sugars can also increase TRG (Hudgins et al., 2000) and the T‐HFHC had more sugar compared to the P‐HFHC, though the difference was not significant. Due to the multifaceted composition of the meal, we are unable to pinpoint the exact component that impacted the TRG and glucose response and wanted to explore the feasibility of administering an entire meal substitute. Still, there were likely overlapping contributions from the polyphenol, fiber content, and plant‐based ingredients, and specific contributions should be explored further. Minimally processed foods alone have been associated with mitigating postprandial TRG and inflammation responses (O'Keefe et al., 2008).

Thorough review papers exist on the mechanisms of action for the reduction of postprandial TRG and glucose using polyphenols (Domínguez Avila et al., 2017; Hanhineva et al., 2010; Kawakami et al., 2021; Kobayashi et al., 2009; Lee et al., 2020) and fiber (Dahl & Stewart, 2015; Katare et al., 2012; Lee et al., 2020; Pasmans et al., 2022). Though not the purpose of this paper, we hypothesize that the primary mechanisms at work are the inhibition of digestive enzymes such as pancreatic lipase and a‐amylase, reduced dietary TRG absorption, and enhanced clearance of TRG (Folwaczny et al., 2021; Hanhineva et al., 2010; Hudgins et al., 2000; Kobayashi et al., 2009; Panth et al., 2020), along with increased GLP‐1, decreased gastric emptying, and decreased starch digestion (Domínguez Avila et al., 2017; Kawakami et al., 2021; Marathe et al., 2013; Pasmans et al., 2022; Slaughter et al., 2002). One of the sources of polyphenols used in the creation of the P‐HFHC was cacao (unprocessed cocoa from the cacao bean). Cacao polyphenols have been shown to inhibit digestive enzymes such as a‐amylase and pancreatic lipase, which control the absorption and digestion of lipids and carbohydrates, and result in the attenuation of plasma TRG and glucose (Gu et al., 2011). Furthermore, cacao polyphenols have been shown to increase insulin and GLP‐1 secretion, thereby managing postprandial glucose responses (Borghouts & Keizer, 2000). Another source of polyphenols was flax seeds. Research by Katare et al. found that flax seeds may suppress phosphoenolpyruvate carboxykinase (PEPCK), a key enzyme in gluconeogenesis (Sciarrillo et al., 2019). Suppression of PEPCK results in decreased glucose production and thus may lower blood glucose (Marathe et al., 2013). Also, research suggests that polyphenols consumed with a high carbohydrate meal increase insulin sensitivity (Aryaeian et al., 2017; Burton‐Freeman, 2010; Edirisinghe et al., 2011). Heightened insulin sensitivity not only improves postprandial glucose but encourages enhanced TRG storage in adipose tissue, which helps to clear TRG from circulation (Burton‐Freeman, 2010; Edirisinghe et al., 2011). Fiber consumption may attenuate the postprandial response through many mechanisms, most notably by increasing GLP‐1 and slowing gastric emptying, which promotes the normalization of blood glucose levels (Marathe et al., 2013) and decreases bile acid reabsorption, thus disrupting fat emulsification and micelle formation (Dahl & Stewart, 2015; Lee et al., 2020; Ou et al., 2001; Pasquier et al., 1996). Dietary fiber has demonstrated potential influences on postprandial metabolic responses through reduced carbohydrate and fat absorption, delayed gastric emptying, and lower insulin secretion. These collectively contribute to improved postprandial glucose and lipid profiles (Yuan et al., 2014). Given that the P‐HFHC meal in our study contained a significantly higher fiber content, it is plausible that dietary fiber may have contributed to the attenuation of postprandial TRG and glucose responses observed. Moreover, the amount and type of fat consumed may also have influenced the postprandial metabolic outcomes. Saturated fats have been associated with increased postprandial lipemia and impaired endothelial function. However, the source of saturated fat may modulate these effects. Plant‐based saturated fats, such as those from coconut oil, may have different metabolic effects compared to animal‐based saturated fats. In the current study, the P‐HFHC meal contained saturated fats from coconut oil, which might have contributed to the observed positive metabolic responses. Research suggests that medium‐chain triglycerides (MCTs) found in coconut oil are metabolized differently than long‐chain triglycerides, potentially leading to lower postprandial triglyceride levels (Kanta et al., 2025; Kasai et al., 2003).

### 4.4 The acute effects of a single nutritional challenge and exercise bout on TRG, glucose, and MLI

The combination of P – HFHC with and without EX was most effective in attenuating PPG and MLI. However, the P‐HFHC and P‐HFHC + EX had moderate effects in reducing TRG, glucose, and MLI AUC compared to T – HFHC. To date, there is limited research investigating the combined effect of a high‐polyphenol, high‐fiber meal, and acute EX on postprandial TRG, glucose, and MLI. Polley et al. studied the effects of tart cherry consumption with and without exercise on TRG following a HFHC. Their findings showed that tart cherry consumption, when combined with exercise, led to a greater reduction in the TRG response (Polley et al., 2019). Interestingly, while our study demonstrated that polyphenol consumption was effective alone, their results indicated that exercise and polyphenol consumption alone were ineffective. In our study, the P‐HFHC had a higher dose of polyphenols than in their study, even with the same bout of exercise. Therefore, in our study, the higher dose used may have allowed the participants to reach the upper limit of the magnitude of attenuation possible. O‐Doherty et al. also investigated the combined effect of exercise and polyphenol intake but only found exercise alone to be effective at reducing the TRG response (O'Doherty et al., 2017). Again, the study design and our P‐HFHC meal may account for the conflicting results when compared to our study. Their participants completed an oral fat tolerance test with 73 g of fat while our participants on average consumed 65 g of fat. On top of that, the phenolic content of their meal was 895 mg, compared to the average of 1154 mg consumed by our participants. Thus, they ingested a smaller amount of polyphenol and a larger amount of fat, which may account for the lack of effect seen. The acute effectiveness of the combined nutritional challenge and exercise bout, compared to either alone, may depend on individual characteristics, the immediate dose of polyphenols relative to fat intake, and the specific exercise parameters.

### 4.5 Experimental considerations

Several limitations within our study that are important to consider. First, while we believe the polyphenols and fiber are the primary ingredients acting to reduce the TRG and glucose response, because our pie was redesigned with different ingredients, we cannot pinpoint the exact mechanisms at play. Also, while the total fat, carbohydrate, and protein content were not statistically different, the P‐HFHC meal substitute contained higher saturated fat. We aimed to keep all ingredients as close as possible to the T‐HFHC using whole food, plant‐based ingredients to maintain similar appearance and texture between the pies. However, it was challenging to perfectly match the nutrient composition. Our objective was to examine the effects of a whole meal substitute, rather than simply supplementation.

Additionally, the participant's heart rate was not recorded during exercise. The heart rate data would allow the postprandial exercise to be classified by intensity. The intensity of the exercise would help to identify the mechanisms of the glucose and triglyceride response. However, a strength of this study was that the energy expenditure was calculated relative to the participants' body mass. Determining the effectiveness of low energy expenditure, irrespective of intensity, was a primary aim to determine whether postprandial responses would be reduced. The applicability of the lower energy expenditure enhanced the true‐to‐life nature of employing a short exercise bout into an individual's typical day.

Next, the time taken by participants to consume each meal was not standardized; instead, the timer for postprandial measurements started upon the final bite of the meal. While this approach aligns with previous studies from our research group (Johnson et al., 2016; Kurti et al., 2017, 2020; Kurti, Wisseman, et al., 2022; Malin et al., 2023; Wisseman et al., 2024), variations in meal consumption speed may have briefly influenced postprandial responses. Finally, this study did not assess menopause status, meaning participants could have been premenopausal, perimenopausal, or postmenopausal. Research indicates that the menopausal transition is linked to elevated fasting and postprandial metabolic responses (Bermingham et al., 2022), with the highest metabolic responses to a meal observed in postmenopausal women (Jackson et al., 2010). Considering the range in the ages of the female participants in the study was 50–69, it is likely our participants were perimenopausal or postmenopausal. Therefore, differences in metabolic responses to the meal and exercise challenge in the present study may have differed between peri‐ and postmenopausal participants.

## 5 CONCLUSIONS

The present study demonstrates that EX expending as low as 25% of the kcals consumed in a HFHC is effective in attenuating PPG and MLI, a significant finding because it is one of the lower energy expenditures we have seen to be effective to date in healthy middle‐aged and older adults. Another key finding of our study is that redesigning a HFHC meal with a high‐polyphenol and fiber content, focusing on a minimally processed product, not only attenuated postprandial TRG responses but also helped participants with fasting glucose in the prediabetic range return to normal glucose levels during the postprandial period. Finally, although the combination of exercise and meal challenge did not result in greater reductions compared to either intervention alone, moderate effect sizes were observed for reductions in TRG, glucose, and MLI AUC. Future studies should examine the mechanisms underpinning how an acute meal and EX can work both independently and synergistically to lower postprandial TRG, glucose, and MLI. Understanding these pathways could offer valuable insights into optimizing dietary and EX interventions to improve metabolic health in middle‐aged and older adults. Therefore, these results will hopefully inform recommendations for middle‐aged and older adults to incorporate minimally processed meals that are high in fiber and polyphenols, as well as establish a routine to engage in postprandial EX.

## FUNDING INFORMATION

Support for the project was provided by the College of Health and Behavioral Studies Collaborative Grant as well as the 4‐VA.

## CONFLICT OF INTEREST STATEMENT

Declaration included. Authors disclose no conflicts of interest.

## ETHICS STATEMENT

The study received IRB approval from James Madison University. All procedures complied with relevant ethical standards. Informed consent was obtained from all participants.

## Data Availability

Data described in the manuscript will be made available upon specific request, application, and approval.
